# Boronic Acid-Modified Magnetic Fe_3_O_4_@mTiO_2_ Microspheres for Highly Sensitive and Selective Enrichment of N-Glycopeptides in Amniotic Fluid

**DOI:** 10.1038/s41598-017-04517-8

**Published:** 2017-07-04

**Authors:** Zhonghua Shi, Liyong Pu, Yueshuai Guo, Ziyi Fu, Wene Zhao, Yunxia Zhu, Jindao Wu, Fuqiang Wang

**Affiliations:** 1 State Key Laboratory of Reproductive Medicine, Department of Biochemistry and Molecular Biology of Nanjing Medical University, Nanjing Maternity and Child Health Care Hospital Affiliated to Nanjing Medical University, Nanjing, Jiangsu 210029 China; 2 Key Laboratory of Living Donor Liver Transplantation, National Health and Family Planning Commission of the People’s Republic of China, Nanjing, China; 3 Department of Liver Transplantation Center, The First Affiliated Hospital of Nanjing Medical University, Nanjing, China

## Abstract

Although mesoporous materials and magnetic materials are used to enrich glycopeptides, materials sharing both mesoporous structures and magnetic properties have not been reported for glycopeptide analyses. Here we prepared boronic acid-modified magnetic Fe_3_O_4_@mTiO_2_ microspheres by covalent binding of boronic acid molecules onto the surfaces of silanized Fe_3_O_4_@mTiO_2_ microspheres. The final particles (denoted as B-Fe_3_O_4_@mTiO_2_) showed a typical magnetic hysteresis curve, indicating superparamagnetic behavior; meanwhile, their mesoporous sizes did not change in spite of the reduction in surface area and pore volume. By using these particles together with conventional poly(methyl methacrylate) (PMMA) nanobeads, we then developed a synergistic approach for highly specific and efficient enrichment of N-glycopeptides/glycoproteins. Owing to the introduction of PMMA nanobeads that have strong adsorption towards nonglycopeptides, the number of N-glycopeptides detected and the signal-to-noise ratio in analyzing standard proteins mixture both increased appreciably. The recovery of N-glycopeptides by the synergistic method reached 92.1%, much improved than from B-Fe_3_O_4_@mTiO_2_ alone that was 75.3%. Finally, we tested this approach in the analysis of amniotic fluid, obtaining the maximum number and ratio of N-glycopeptides compared to the use of B-Fe_3_O_4_@mTiO_2_ alone and commercial SiMAG-boronic acid particles. This ensemble provides an interesting and efficient enrichment platform for glycoproteomics research.

## Introduction

As a biologically broad and significant post-translational modification, protein glycosylation is involved in many physiological activities and disease states, such as protein folding, cell division, intracellular secretion, inflammation, and congenital disorders^1–4^. Moreover, it has been discovered that more than half cancer biomarkers are glycosylated peptides or proteins^5^. In the present glycoproteome research, the discovery of glycosylation site occupancy and identification of glycan heterogeneity at each glycosite have been largely undertaken by the use of mass spectrometry (MS) or tandem MS/MS^6, 7^. Glycoproteins and glycopeptides in real samples are much lower in amount compared to nonglycosylated ones, and besides, their ionization efficiency is rather poor, both of which result in negative signal suppression in MS analysis. Therefore, in order to obtain high-resolution profiling of endogenous glycoproteins in serum or tissues, use of efficient strategies for specific isolation and enrichment of the targets is indispensable.

For the past years, methods for glyco-specific enrichment divide into several categories by means of different mechanisms, including lectin affinity^8, 9^, size exclusion^10^, hydrazide chemistry^11, 12^, hydrophilic interaction^13, 14^, and boronic acid-derived matrixes^15–19^, with the last one receiving more attention recently. With phenylboronic acids typically employed, the boronic acid moiety can form cyclic ester with *cis*-diol group of glycoconjugates in an alkaline medium and at acidic pH the ester dissociates^19^, making boronic acid a unique ligand for reversibly collecting and detaching glycopeptides. Besides, this method isolates both N- and O-glycopeptides in an unbiased manner, thus complementing some limitations of other methods, such as biased glycol-enrichment with respect to lectin affinity and hydrazide chemistry, and insufficient selectivity and recovery related to hydrophilic interaction. Several types of materials have been developed to conjugate boronic acid groups for glycopeptide analyses, including agarose resin, mesoporous silica^15^, polymer particles^16^, magnetic particles^18–24^, carbon nanotubes^25^, and graphene oxide^26^. Each type of material has its own merit. Boronic acid-agarose resin has already been commercialized, boronic acid-mesoporous silica shows large pore volume and pore size, boronic acid-magnetic particles display facile separation by external magnetic field, and boronic acid-carbon nanotubes or graphene oxide possess exceptionally large specific surface area and high density of boronic acid groups.

We wish to confer mesoporous structure combined with magnetic property to the boronic acid-modified composite for the enrichment of glycopeptides. Mesoporous structure can bring about large surface area and tunable pore size for the flux of targets, and meanwhile, the response to magnetic field enables convenient separation from the complex matrixes in real applications. To our best knowledge, there is no report on the enrichment of glycopeptides by such kind of material. Recently, Ma *et al*. developed a method to prepare magnetic core/shell Fe_3_O_4_@mTiO_2_ microspheres for highly efficient enrichment of phosphopeptides^27^. The microsphere owns the features which meet well with what we entail, including a mesoporous crystalline TiO_2_ layer ensuring a large absorption capacity and a high mass transport efficiency, and a Fe_3_O_4_ colloidal cluster core with excellent magnetic response. After post-functionalization with boronic acid group, we anticipate the composite to capture and separate glycopeptides efficiently. In order to obtain a better selectivity, we employed a synergistic strategy by adopting conventional PMMA nanobeads as the second enriching material to reduce the effect of nonglycopeptides^28^. Finally, we tested the method in the glycopeptides analysis of human amniotic fluid samples with remarkable results.

## Methods

### Materials

Iron(III) chloride hexahydrate (FeCl_3_ · 6H_2_O), ammonium acetate (NH_4_Ac), trisodium citrate dehydrate, ethylene glycol, anhydrous ethanol, aqueous ammonia solution (25%), sodium borohydride (NaBH_4_), methyl methacrylate (MMA), and titanium(IV) butoxide were purchased from Shanghai Chemical Reagents Company. 3-aminopropyl-trimethoxysilane (APTMS) and 3-formylbenzeneboronic acid (FBBA) were purchased from JK Chemical Company. Horseradish peroxidase (HRP, 98%), myoglobin (MYO, 95%), fetuin (98%), ammonium bicarbonate (ABC, 99.5%), dithiothreitol (DTT, 99%), acetone (99.9%), iodoacetamide (IAA, 99%), acetonitrile (ACN, 99.9%), trifluoroacetic acid (TFA, 99.8%), 2,5-dihydroxybenzoic acid (DHB, ≥99.5%) were obtained from Sigma-Aldrich. Sequencing grade modified trypsin was purchased from Promega. Peptide-N-Glycosidase F (PNGaseF) was obtained from New England Biolabs. All these reagents were used as received without further purification. Deionized water (18.2 M cm) was used throughout the experiments.

### Preparation of Core/Shell Fe_3_O_4_@mTiO_2_ Microspheres

The magnetic core/shell Fe_3_O_4_@mTiO_2_ microspheres were prepared by three steps according to the literature^27^. (1) Prepare citrate-stabilized Fe_3_O_4_ clusters. Nearly 1.35 g of FeCl_3_ · 6H_2_O, 3.8 g of NH_4_Ac, and 0.4 g of sodium citrate were added into 70 mL of ethylene glycol. The mixture turned to a homogeneous black solution under magnetic stirring at 170 °C for 1 h, which was then transferred into a Teflon-lined stainless-steel autoclave (100 mL capacity) and maintained at 200 °C for 16 h. After cooled down to room temperature (RT), the product was obtained by magnetic precipitation, and washed by water (three times) and ethanol (two times) until the supernatant became colorless. Finally the product was stored in ethanol at a concentration of 5 mg/mL. (2) Encapsulate Fe_3_O_4_ colloids by a layer of TiO_2_, generating core/shell Fe_3_O_4_@TiO_2_ microspheres. 10 mL of the as-prepared Fe_3_O_4_ colloids was mixed with 80 mL of ethanol, 30 mL of acetonitrile, and 0.5 mL of NH_3_ · H_2_O. After ultrasound treatment for several minutes, the mixture was added with 1 mL of titanium butoxide under mechanic stirring, and the reaction continued for about 1.5 h. The products were collected by repeated cycles of magnetic separation and washing with acetonitrile and ethanol. (3) Obtain Fe_3_O_4_@mTiO_2_ microspheres by hydrothermal treatment of the Fe_3_O_4_@TiO_2_ microspheres to form mesoporous TiO_2_ shell. The product obtained in the second step was ultrasonically dispersed in ethanol/H_2_O (40 mL/20 mL), followed by the addition of 3 mL of NH_3_ · H_2_O. It was transferred into a Teflon-lined stainless-steel autoclave (100 mL capacity) and maintained at 160 °C for 20 h. After cooled down to RT, the product was obtained by magnetic precipitation, washed with ethanol several times, and dried at 60 °C. Such brick-colored material was made up of Fe_3_O_4_@mTiO_2_ microspheres.

### Preparation of Boronic Acid-Modified Fe_3_O_4_@mTiO_2_ Microspheres

**Figure 1:**
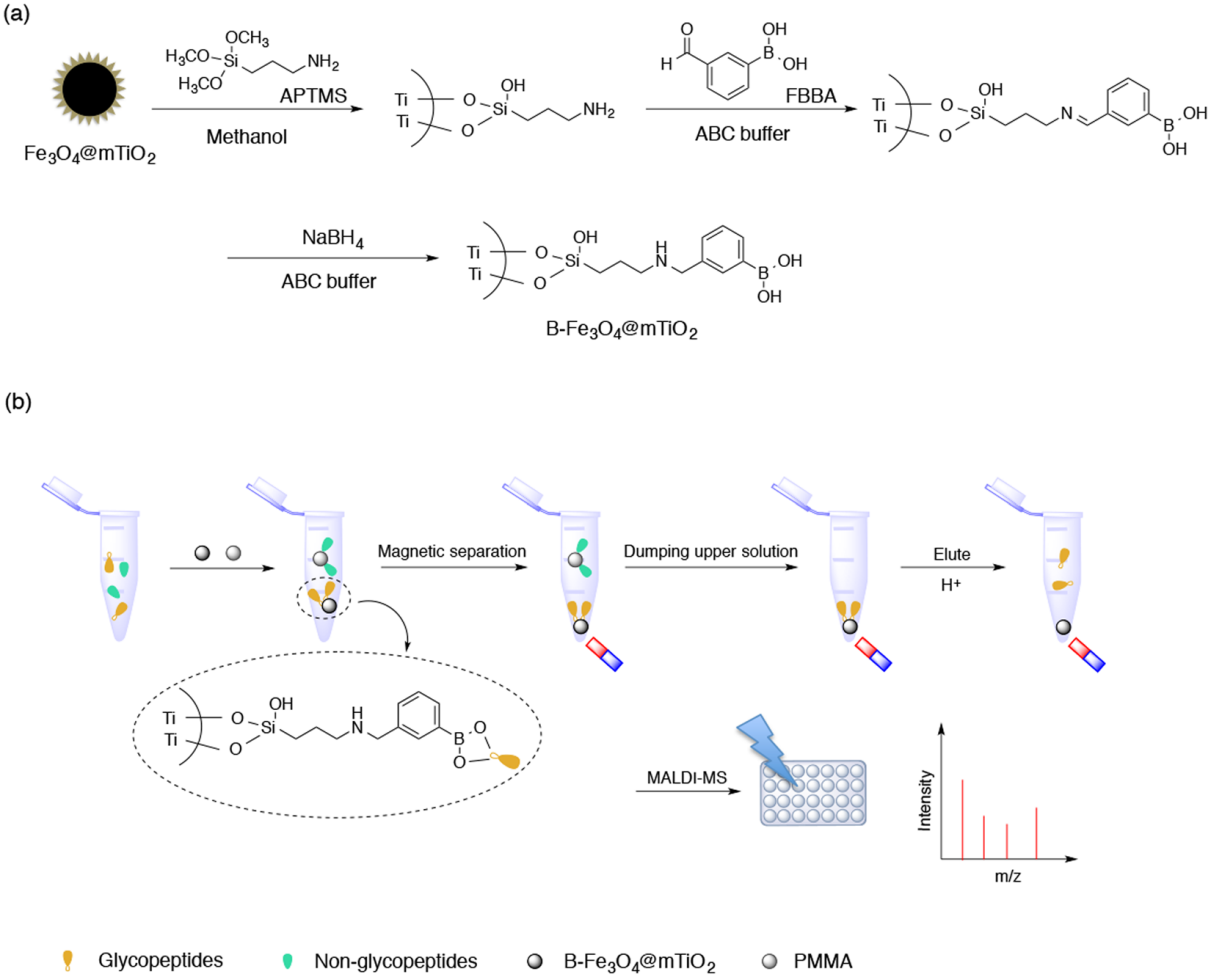
Graphic illustration of (**a**) the preparation steps for B-Fe_3_O_4_@mTiO_2_ and (**b**) the procedure for the detection of N-glycopeptides using a combination of B-Fe_3_O_4_@mTiO_2_ and PMMA.

The grafting of boronic acid was acheived *via* three steps, as demonstrated in Fig. 1a. (1) Treat the Fe_3_O_4_@mTiO_2_ microspheres with APTMS. 50 mg of Fe_3_O_4_@mTiO_2_ microspheres was redispersed by sonication in 40 mL of methanol. Then 0.2 mL of APTMS was added, heated, and refluxed at 80 °C for 4 h. The product was sufficiently rinsed with methanol to remove any remaining APTMS. Finally it was suspended in 40 mL of ABC buffer (10 mM). (2) Covalent binding of FBBA. 20 mg of FBBA was dissolved completely in 1 mL of ethanol, and it was added to the APTMS-treated Fe_3_O_4_@mTiO_2_ microspheres for 2 h of reaction at 65 °C with vigorous shaking. Afterwards, the product was collected and rinsed with ethanol several times to remove excess FBBA moiety. (3) Reduce the formed Schiff base with NaBH_4_. 40 mL of ABC buffer (10 mM) containing the FBBA-grafted magnetic microspheres was added with 20 mg of NaBH_4_ and reacted overnight at RT. After rinsing with ethanol and drying at 60 °C, we obtained the final product boronic acid-modified Fe_3_O_4_@mTiO_2_ microspheres, denoted as B-Fe_3_O_4_@mTiO_2_.

### Preparation of PMMA Nanobeads

The PMMA nanobeads were prepared according to a previous method^29^. 5 g of MMA monomer and 0.15 g (NH_4_)_2_S_2_O_8_ were added to 65 mL of water and reacted at 75 °C for 4 h with magnetic stirring. The obtained PMMA nanobeads were centrifuged at a speed of 6,000 rpm, sufficiently rinsed with water, and dried at 80 °C.

### Sample Preparation

Amniotic fluid samples were obtained from Nanjing Maternity and Child Health Care Hospital with written consent and approval of the ethics board. The experimental protocols were approved by Nanjing Medical University. All methods were performed in accordance with the relevant guidelines and regulations. All amniotic acid specimens (8–10 mL) from women at 16–18 weeks of gestation, carrying out prenatal diagnosis mostly due to advanced maternal age ranging from 30 to 45 years, were centrifuged at 12,000 rpm for 15 min at 4 °C to remove insoluble debris after thawing on ice. Afterwards, the cell-free supernatants were vacuum-dried with a SpeedVac system (RVC 2–18, Marin Christ, Osterode, Germany). Then they were added with acetone (1:4, V/V), briefly vortexed, and incubated at −20 °C for 60 min. After centrifugation, the sediments were collected and dissolved in ABC buffer (50 mM), and the protein concentrations were measured by using the Pierce BCA protein assay kit (Rockford, IL, USA). Finally, they were stored at −80 °C for further LC-MS/MS analysis.

### Digestion of Standard Proteins and Protein Mixture from Amniotic Fluid

The standard proteins, i.e. HRP, MYO, and fetuin, were each dissolved in ABC buffer (50 mM) at 1.0 mg/mL, and denatured at 100 °C for 5 min. After cooled down, trypsin was added at an enzyme-to-protein ratio of 1: 40 (w/w) for hydrolysis overnight at 37 °C, respectively.

For the digestion of amniotic fluid, proteins of amniotic fluid (1 μL) were reduced with 10 mM of DTT for 30 min at 60 °C and alkylated with 20 mM of IAA for 30 min at 37 °C in dark. Then trypsin digestion was applied to the sample, as described above. Finally, desalting of the sample was conducted on C18 columns before it was stored at −20 °C for further use.

### Synergistic Enrichment of N-Glycopeptides

The procedure is illustrated in Fig. 1b. The digestion products of standard proteins and peptides mixture from amniotic fluid were diluted with ABC buffer (50 mM) to 200 μL, followed by the addition of 10 μL of B-Fe_3_O_4_@mTiO_2_ suspension (10 mg/mL) and 40 μL of PMMA nanobeads (10 mg/mL). After shaking at RT for 1 h, a magnet was used to separate the glycopeptides-captured B-Fe_3_O_4_@mTiO_2_ microspheres, and without rinsing, the glycopeptides release was performed using 20 μL of elution buffer (ACN/H_2_O/TFA, 20:79:1 by volume) at RT for 1 h prior to the subsequent MS analysis.

### MALDI-TOF/TOF MS Analysis

For the analysis of enriched glycopeptides, 1 μL of eluate was deposited on a MALDI plate, and then 1 μL of DHB matrix (12.5 mg/mL in ACN/H_2_O/TFA, 50:49.9:0.1 by volume) was spotted onto 600 μm anchorchips (Bruker Daltonics, Bremen, Germany). The Bruker peptide calibration mixture was spotted for external calibration. MALDI-TOF/TOF MS was carried out on a time-of-flight Ultraflex Extreme mass spectrometer (Bruker Daltonics, Bremen, Germany). Peptide mass maps were acquired in positive reflection mode, averaging 800 laser shots per spectrum. Resolution was 15000–20000. The Bruker calibration mixtures were used to calibrate the spectrum to a mass tolerance within 0.1 Da. Each acquired mass spectrum (m/z range 1000–5000) was processed using the software FlexAnalysis v.2.4 supplied by Bruker Daltonics. The peak detection algorithm was SNAP (Sort Neaten Assign and Place), signal-to-noise (*S*/*N*) threshold was 3, and the quality factor threshold was 50.

### Nano-Liquid Chromatography Tandem Mass Spectrometry (Nano-LC*−*MS/MS) Analysis of Glycopeptides

The eluate containing the enriched glycopeptides was lyophilized and then redissolved in ABC buffer (50 mM). Deglycosylation was performed by the addition of PNGase F (1 μL) into the peptides solution prepared from digestion of crude proteins (1 mg), maintaining at 37 °C for 16 h. The deglycosylated peptides were then subject to nano-LC-MS/MS analysis. The labeled deglycosylated peptides were applied on the LTQOrbitrap instrument (Thermo Fisher, USA) equipped with a Waters Nano ACQUITY UPLC system via a nanospray source for data acquisition. The LC-MS/MS was operated in positive ion mode. The analytical method was set at a linear gradient from 0 to 60% of ACN in 150 min, and flow rate of 200 nL/min. For analysis of amniotic fluid from human placenta, one full MS scan was followed by five MS/MS scans on those five highest peaks respectively.

### Database Search

The raw data derived from the LC-MS/MS analyses were processed by MaxQuant software (version 1.5.2.8)^30^, and searched against the reference human protein sequences from the UniProt database (Release 2015_10; 70071 sequences)^31^. The parameters for the MaxQuant search were as follows: enzyme (trypsin/P), missed cleavages (2), minimum peptide (6), fixed modification (carboxyamidomethylation, C), variable modifications: deamidation 18 O (N) and oxidation (M), peptide tolerance (6 ppm), MS/MS tolerance (0.5 Da). The false discovery rate (FDR) of the identification was estimated by searching against the database with the reversed protein sequences. The site, peptide and protein FDRs were all set at 0.05. To further obtain reliable results, only glycosylated sites with the canonical sequence motif (N-!P−S/T/C)^32^ and a minimum localization probability of 0.5 were reported.

### Characterization

Transmission electron microscopy (TEM) was carried out on a JEOL-2100F transmission electron microscope operating at 200 kV. Scanning electron microscopy (SEM) was carried out on a Zeiss Supra 40 field-emission scanning microscope at an acceleration voltage of 5 kV. N_2_ adsorption-desorption analyses were conducted using a Micrometritics ASAP 2020 accelerated surface area analyzer at 77 K, using Barrett-Emmett-Teller (BET) calculations for the surface area. Before measurements, the samples were degassed in a vacuum at 120 °C for at least 6 h. Fourier transform infrared (FTIR) spectra were measured on a Bruker Vector-22 FTIR spectrometer from 4000 to 400 cm^−1^ at room temperature. Power X-ray diffraction (PXRD) data were recorded on a Philips X’Pert PRO SUPER X-ray diffractometer equipped with graphite-monochromatized Cu K**α** radiation. X-ray photoelectron spectroscopic (XPS) study was performed on an ESCALAB 250 spectrometer (Thermo-VG Scientific). The magnetization curve was measured with a superconducting quantum interference device (SQUID) magnetometer (Quantum Design MPMS XL).

## Results and Discussion

### Synthesis and Characterization of B-Fe_3_O_4_@mTiO_2_ microspheres

**Figure 2:**
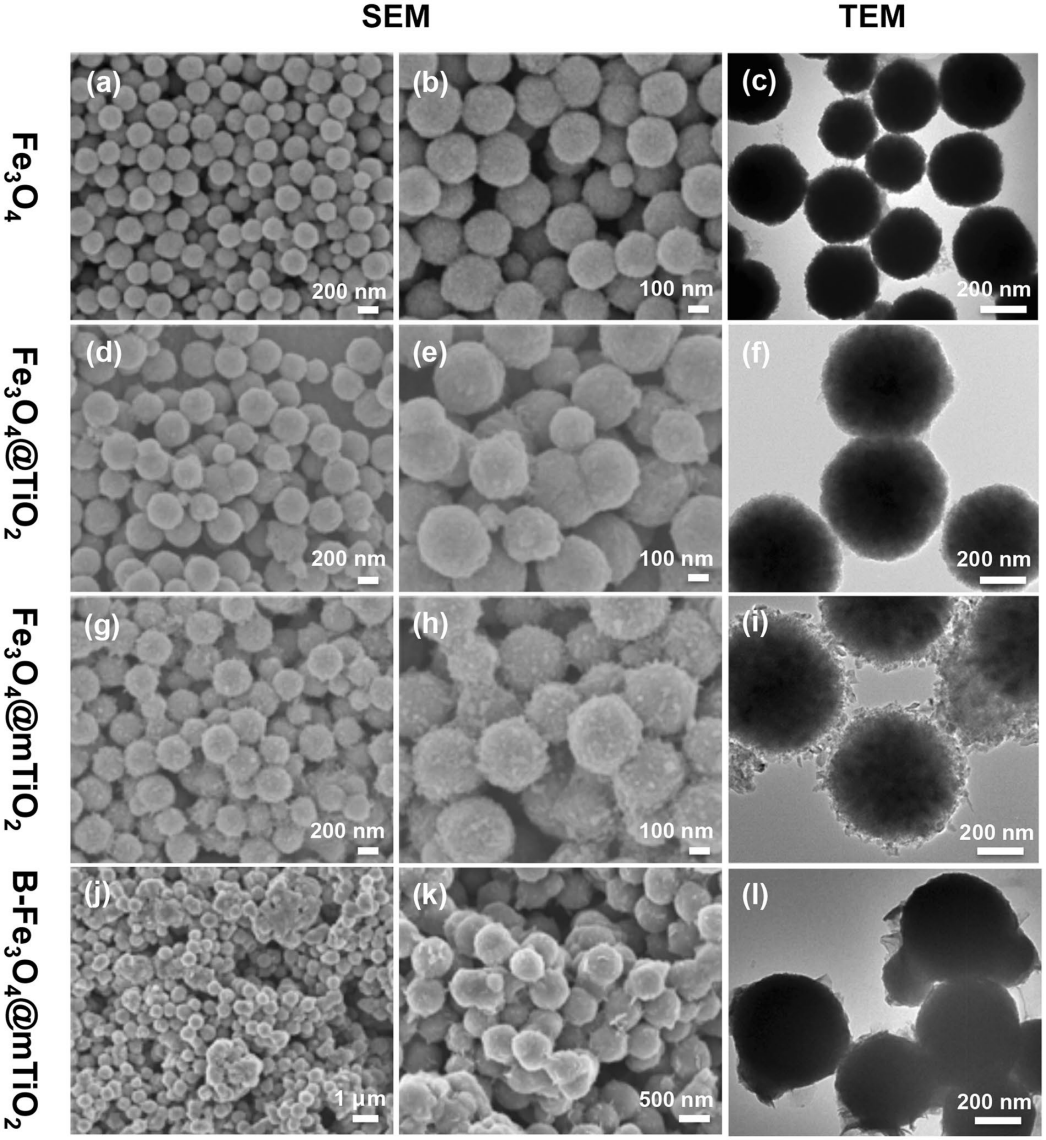
SEM and TEM images (**c,f,i,l**) of Fe_3_O_4_ (**a–c**), Fe_3_O_4_@TiO_2_ (**d–f**), Fe_3_O_4_@mTiO_2_ (**g–i**), and B-Fe_3_O_4_@mTiO_2_ particles (**j–l**).

It was found that both the volume ratio of ethanol to water and the amount of ammonium were critical to pore evolution of the TiO_2_ shell. To acquire a relatively large pore for the flux of glycopeptides, the volume ratio of ethanol to water was set at 40:20 and 3 mL of NH_3_ · H_2_O was applied. We analyzed the size and morphology of the particles by SEM and TEM. From the SEM images (Fig. 2a,d,g), we observed an obvious increase of size for the Fe_3_O_4_@TiO_2_ microspheres compared to bare Fe_3_O_4_, and little change of size after their transformation to Fe_3_O_4_@mTiO_2_. From the enlarged SEM images (Fig. 2b,e,h), we could observe that the Fe_3_O_4_@TiO_2_ microspheres had smooth surfaces as those for the bare Fe_3_O_4_ particles, suggesting a homogeneous shell of amorphous TiO_2_ around the magnetic core. This was confirmed by the PXRD study, showing no characteristic TiO_2_ crystal peaks (Fig. S1). After hydrothermal reaction, the surfaces of those Fe_3_O_4_@mTiO_2_ particles became uneven, which was owing to the formation of crystalline TiO_2_ and generation of mesoporous structure. This was further demonstrated by TEM images (Fig. 2c,f,i), with a shell of TiO_2_ crystals with sizes falling within 20–30 nm surrounding the core for the Fe_3_O_4_@mTiO_2_ microspheres. PXRD also showed the appearance of a new diffraction peak near 25° corresponding to the (101) plane of anatase-phase TiO_2_ (Fig. S1)^33^. After modification with boronic acid, the SEM images (Fig. 2j,k) showed a little aggregation of particles, and the TEM result (Fig. 2l) revealed a layer of organic substance adsorbed onto the surfaces of the mesoporous particles.

**Figure 3:**
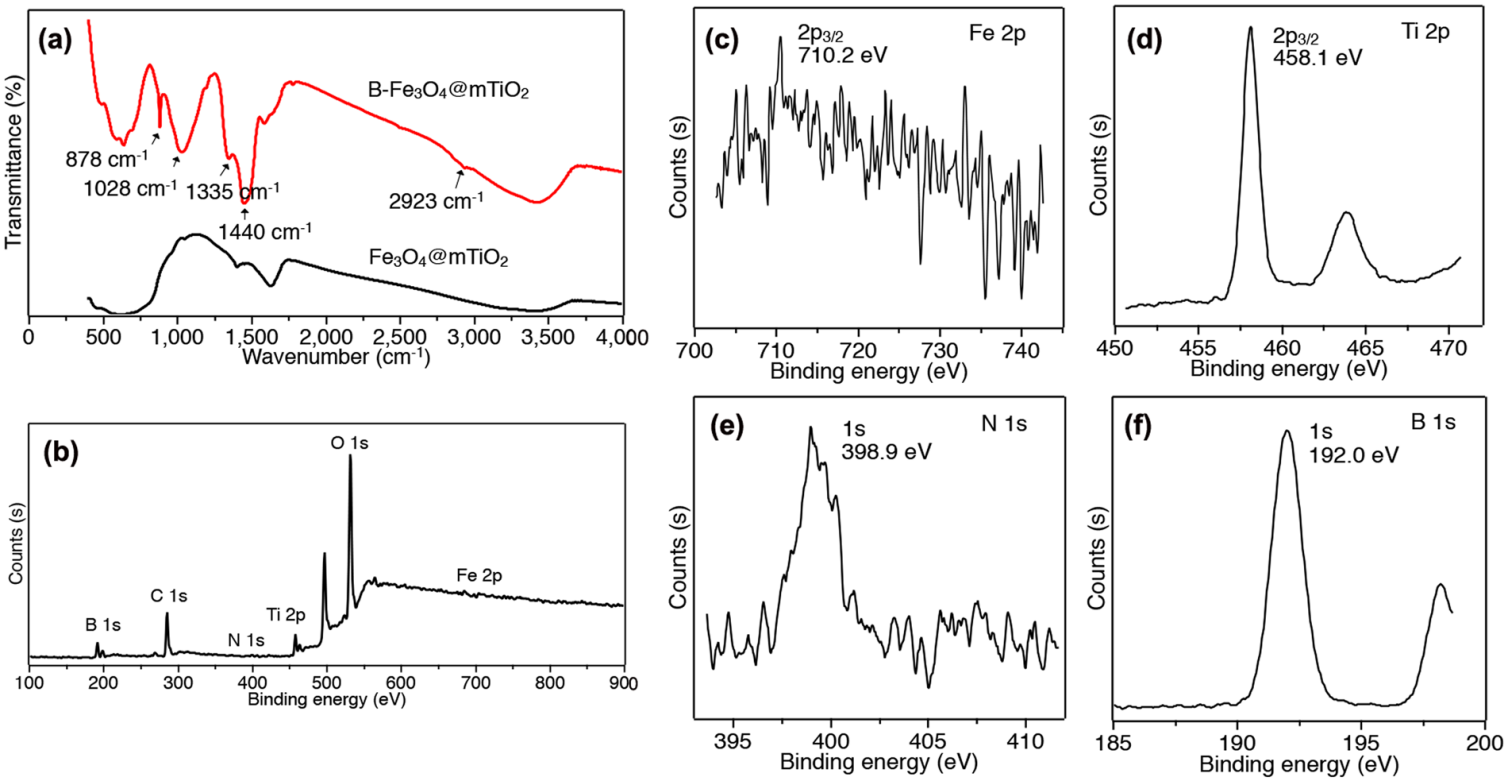
FTIR (**a**) and XPS survey spectra (**b**) of B-Fe_3_O_4_@mTiO_2_. XPS spectra with respect to Fe (**c**), Ti (**d**), N (**e**), and B (**f**).

We identified the successful modification of boronic acid by means of FTIR and XPS. Compared to the Fe_3_O_4_@mTiO_2_ microspheres, the sample after reactions possessed several new bands in its FTIR spectrum, as shown in Fig. 3a. A weak band at 2923 cm^−1^ was ascribed to the -CH_2_ absorption from the silane agent APTMS. The strong peaks at 878 and 1440 cm^−1^ were attributed to benzene ring vibrations from FBBA, and the presence of an obvious peak at 1335 cm^−1^ clearly corresponded to the B-O stretching^28^. In addition, another strong peak at 1028 cm^−1^ should be caused by the absorption of Si-O band^34^. The survey XPS spectrum exhibited the presence of C, N, O, B, Fe, and Ti in the sample, the binding energy (BE) for Fe 2p3/2 locating at 710.2 eV and the BE for Ti 2p3/2 at 458.1 eV (Fig. 3b–d). The low intensity of the Fe 2p signal was due to core encapsulation by TiO_2_. The BE for N 1 s was centered at 398.9 eV obviously (Fig. 3e), implying the adsorption of APTMS. Lastly the BE for B 1 s was observed at 192.0 eV (Fig. 3f), which was a solid proof for the binding of FBBA. From the above results, we can say that the modification of Fe_3_O_4_@mTiO_2_ microspheres by boronic acid is successful.

**Figure 4:**
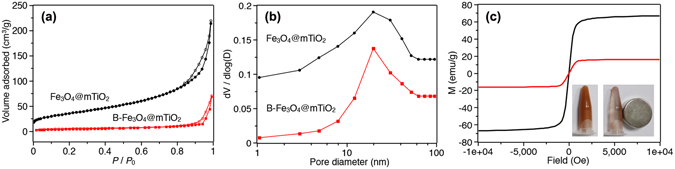
N_2_ adsorption-desorption isotherms (**a**) and pore-size distribution curves (**b**) for Fe_3_O_4_@mTiO_2_ and B-Fe_3_O_4_@mTiO_2_. (**c**) Room-temperature magnetization curves of Fe_3_O_4_ (black line) and B-Fe_3_O_4_@mTiO_2_ (red line). Inset shows magnetic response of B-Fe_3_O_4_@mTiO_2_ to a magnet.

We further investigated the mesoporous character of the microspheres before and after boronic acid modification by N_2_ sorption analyses. It clearly showed that both particles manifested typical type IV gas sorption isotherms (Fig. 4a), hence implying that the mesoporous structure was not destroyed by the post-modification. Using the BET model for calculations, the specific surface area dropped from 146.0 m^2^/g for Fe_3_O_4_@mTiO_2_ to 19.6 m^2^/g for B-Fe_3_O_4_@mTiO_2_, and meanwhile, the pore volume decreased from 0.31 to 0.11 cm^3^/g appreciably. However, from the pore size distribution curves (Fig. 4b), the sizes of the pore cavities kept barely changed, centered at 19.6 and 19.5 nm, respectively. The reduced surface area and pore volume should be explained by the organic grafting in the particles, which blocked some of the tiny slits between the neighboring TiO_2_ crystals in the shell. This was also observed in the boronic acid-functionalized mesoporous silica^15^. Despite the reduction in surface area and pore volume, the relative large pore size was still favorable for the permeation of targeted glycopeptides during the enrichment process. Then the magnetic property was assessed, as shown in Fig. 4c. The magnetic hysteresis curve showed the saturation magnetization (Ms) value decreasing from 67 emu/g for the bare Fe_3_O_4_ to 16 emu/g for the B-Fe_3_O_4_@mTiO_2_, which was caused by the surface modification. Despite the drastic reduction of Ms value, the superparamagnetic feature of the B-Fe_3_O_4_@mTiO_2_ microspheres expedited their efficient separation in 30 s with a magnet. Such a fast response was beneficial to practical applications.

### Specific Enrichment of N-Glycopeptides by a synergy of B-Fe_3_O_4_@mTiO_2_ Microspheres with PMMA Nanobeads

**Figure 5:**
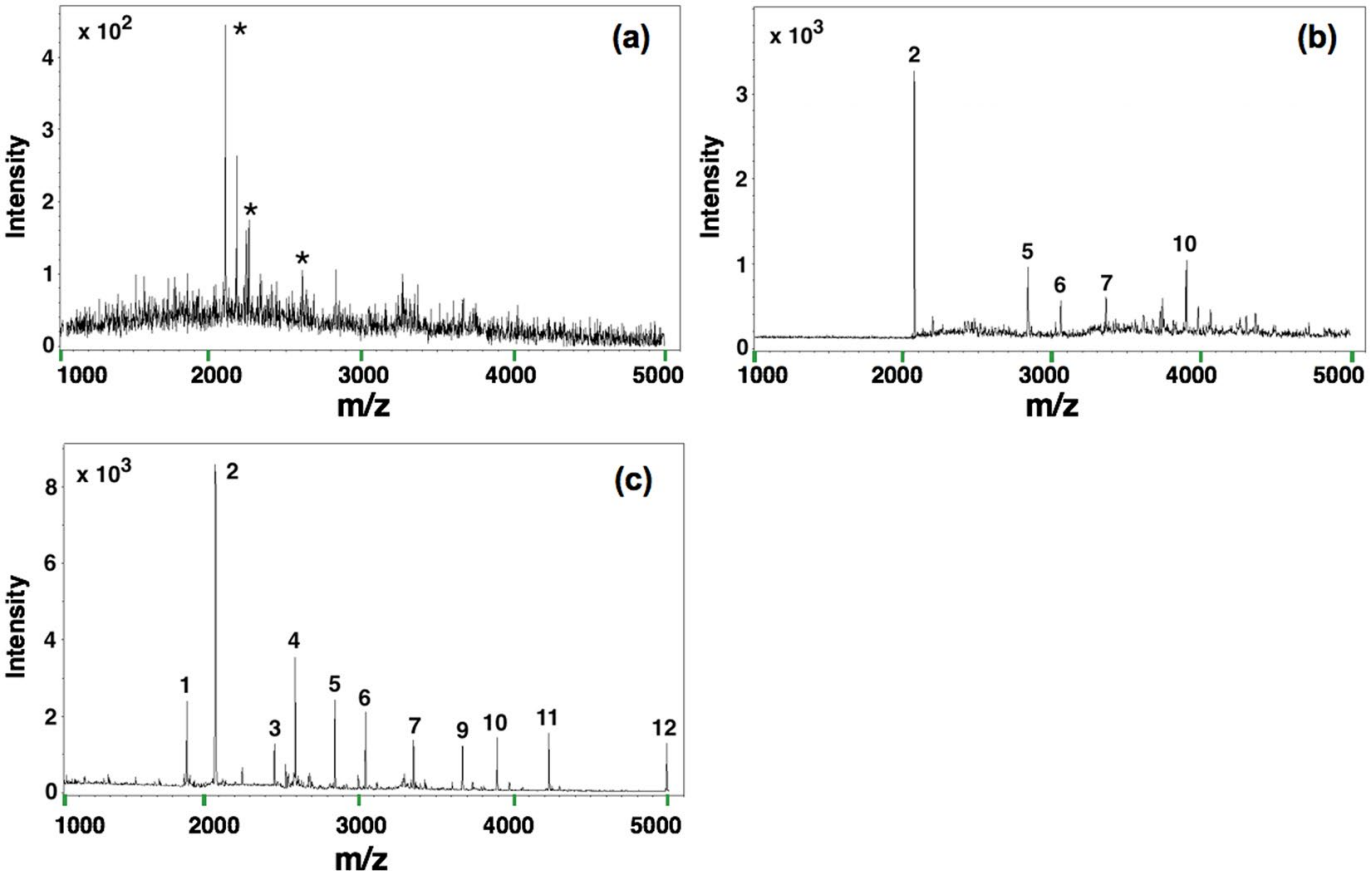
MALDI-TOF/TOF mass spectra of the tryptic digest of 0.1 ng/μL HRP: (**a**) without enrichment, (**b**) after enrichment by B-Fe_3_O_4_@mTiO_2_ microspheres, and (**c**) after enrichment by B-Fe_3_O_4_@mTiO_2_ microspheres and PMMA nanobeads. Note that asterisk marks label peaks of nonglycopeptide, and Arabic numbers label peaks of glycopeptide.

The enrichment of N-glycopeptides for the B-Fe_3_O_4_@mTiO_2_ particles was first tested with the tryptic digest of HRP, a standard glycoprotein, by MALDI-TOF/TOF MS. In the absence of enriching material, only signals for nonglycopeptides were detected (Fig. 5a), while after enrichment by B-Fe_3_O_4_@mTiO_2_, five peaks were identified corresponding to N-glycopeptides from the digestion of HRP (Fig. 5b). This detected number of glycopeptides is comparable with those by using enriching materials such as FDU-12-GA (5) and Fe_3_O_4_@SiO_2_-APB (3)^15, 28^, but much less than those by using core-satellite composite (17) and APBA-MCNTs (21)^23, 25^. We assume that this difference originates from the difference in experimental conditions, including content of boronic acid, instrument, and concentration of HRP used, etc. Apart from the increase in the number of N-glycopeptides detected, the use of B-Fe_3_O_4_@mTiO_2_ also lowered the intensities from the nonglycopeptides, thereby improving the signal-to-noise (*S*/*N*) ratio of the N-glycopeptides greatly. This result demonstrated the excellent specificity of the B-Fe_3_O_4_@mTiO_2_ to N-glycopeptides.

To acquire more number of N-glycopeptides signals, we employed PMMA nanobeads as a second material for enriching nonglycopeptides. With the introduction of PMMA, the number of detected N-glycopeptides increased to 11 (Fig. 5c). Not only this, the peak intensities were much higher with a cleaner background in the mass spectrum, suggesting that the *S*/*N* ratio could be dramatically increased. Peak 3 at m/z = 2445 was chosen to evaluate the sensitivity of the method. At the present concentration of HRP (0.1 ng/μL), the *S*/*N* ratio was larger than 100, implying that the limit of detection for our method was at the level of 10 pg/μL. This value is comparable with those obtained by using core-satellite composite, Fe_3_O_4_@SiO_2_-APB, and APBA-MCNTs^23, 25, 28^. All the results revealed the striking advantage of the synergistic enrichment strategy. The detailed sequence information of all the glycopeptides identified is listed in Table S1. A comparable sensitivity of detection was also observed with fetuin as the standard N-glycoprotein (Fig. S2). This suggests that N-glycopeptides from other model proteins can be also enriched by a combination of the B-Fe_3_O_4_@mTiO_2_ microspheres and PMMA nanobeads.

**Figure 6:**
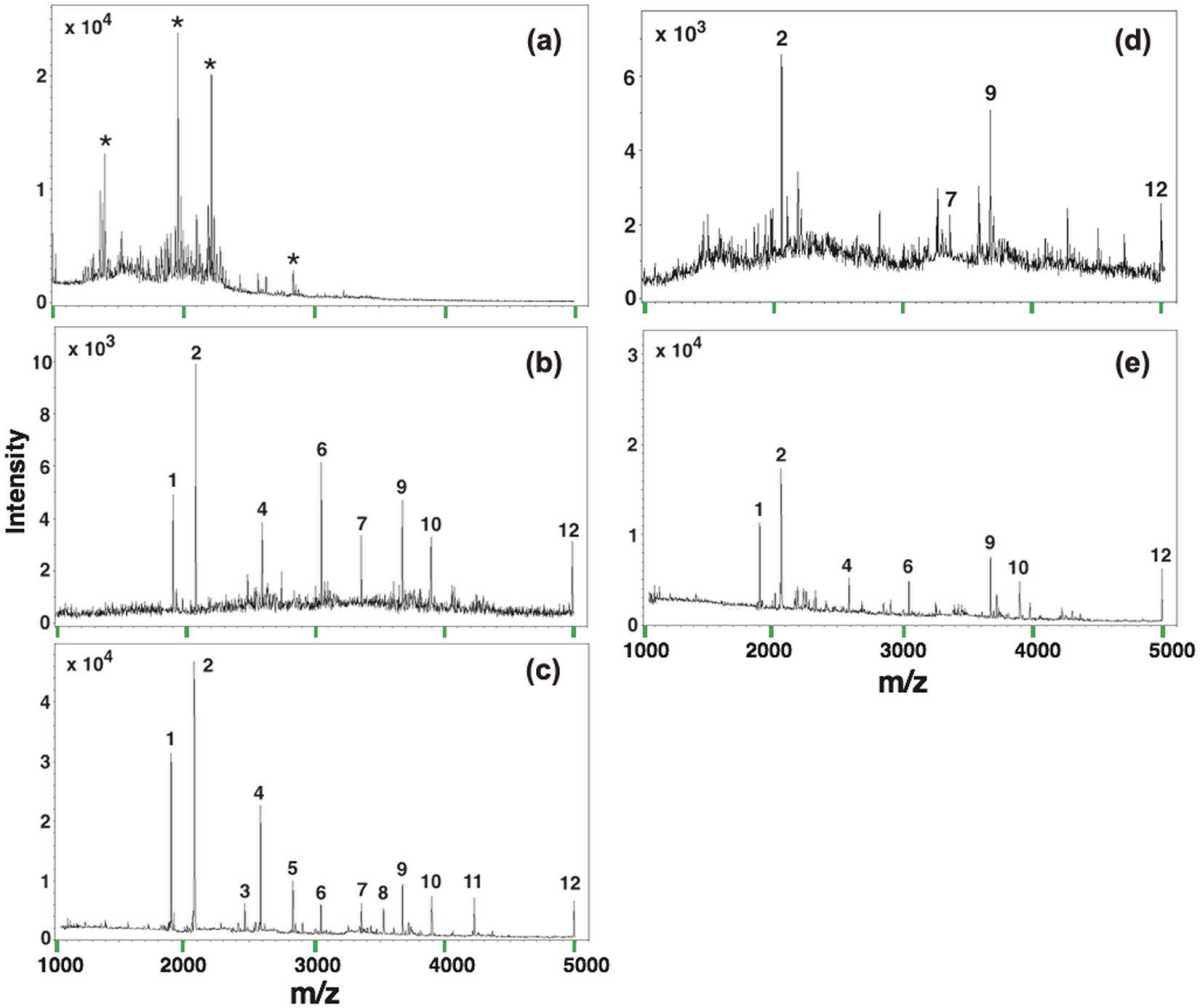
MALDI-TOF/TOF mass spectra of the tryptic digest mixture of HRP and MYO (n/n = 1/100): (**a**) without enrichment, (**b**) after enrichment by B-Fe_3_O_4_@mTiO_2_ microspheres, (**c**) after enrichment by B-Fe_3_O_4_@mTiO_2_ microspheres and PMMA nanobeads, (**d**) after enrichment by commercial SiMAG-boronic acid, and (**e**) after enrichment by SiMAG-boronic acid and PMMA nanobeads. Note that asterisk marks label peaks of nonglycopeptide, and Arabic numbers label peaks of glycopeptide.

We then evaluated the selectivity of the method by mixing the standard N-glycopeptides (from HRP) with the standard nonglycopeptides (from MYO) at a molar ratio 1:100 of HRP to MYO. Direct analysis generated only signals for nonglycopeptides with complicated background in the spectrum (Fig. 6a). By the use of B-Fe_3_O_4_@mTiO_2_, 8 peaks for N-glycopeptides were detected, and the interfering peaks related to nonglycopeptides vanished completely (Fig. 6b). After the introduction of PMMA nanobeads, 4 more peaks were detected and a much larger *S*/*N* ratio was also expected (Fig. 6c). This is owing to the unspecific adsorption of nonglycopeptides by PMMA nanobeads to create more opportunities for B-Fe_3_O_4_@mTiO_2_ to interact with targeted peptides. Besides, the traditional washing step was avoided for this approach, thus minimizing the loss of glycopeptides to the least. The binding capacity of this method was measured to be 120 mg/g (Fig. S3). Next the performance of B-Fe_3_O_4_@mTiO_2_ was compared to that of commercial particles SiMAG-boronic acid. Under identical conditions, there were 4 peaks related to N-glycopeptides in the spectrum with using bare SiMAG-boronic acid (Fig. 6d). By combining PMMA nanobeads, 3 more peaks for glycopeptides were detected with a higher sensitivity as well (Fig. 6e). Taken together, we can expect better performance of detection by employing the synergistic enrichment method and the enriching capability of B-Fe_3_O_4_@mTiO_2_ is superior to that of commercial SiMAG-boronic acid particles.

We further investigated the recovery of N-glycopeptides. A pre-prepared tryptic digested HRP was divided into two equivalent parts. One was treated with PNGaseF in H_2_^18^O to release the glycans, and the other involved capturing of the glycopeptides with B-Fe_3_O_4_@mTiO_2_, eluting them, and then treating with PNGaseF in H_2_^16^O. Through mixing the two parts, we could profile the products with MS to comparatively study the abundances of the glycopeptides from different oxygen isotopes according to peak areas. Inferred from the MALDI-TOF results (Fig. S4 and Table S2), the recovery of N-glycopeptides by the synergistic method was determined to be 92.1%, much improved than from B-Fe_3_O_4_@mTiO_2_ alone which was 75.3%. This recovery value exceeds that enriched by a combination of Fe_3_O_4_@SiO_2_-APB with PMMA (90%)^28^, and thus it makes the best among the boronic acid-based methods for enriching glycopeptides to date.

To test the applicability of the new method, amniotic fluid was examined as a model biological sample for identifying the N-glycopeptides and N-glycoproteins. The quantitative and qualitative analysis of amniotic acid may help to identify patients who will develop pregnancy complications or to discover fetal-disease specific markers^35^. After pretreatment, the proteins in the sample were trypsin-digested and incubated with enriching materials for capturing glycopeptides. Three replications using three enriching ensembles were performed to test the applicability and optimize the usage of the presented material in the enrichment of glycopeptides. First, a total of 126 N-linked glycopeptides corresponding to 97 glycoproteins were identified (Table S3). Second, the maximum number and ratio of N-glycopeptides detected were obtained by the synergistic enrichment method (Table S4). 9 more N-glycopeptides and 4 more N-glycoproteins were identified by the combined materials than by the B-Fe_3_O_4_@mTiO_2_ alone. We also found that the ratio of enriched glycopeptides obtained by the combined materials was significantly higher than SiMAG-boronic acid using Fisher’s exact test (Fold change = 1.5 and *P* value = 0.04). Besides, the ratio was not significantly improved (*P* = 0.56) compared to that of using B-Fe_3_O_4_@mTiO_2_ alone, but we did identify more N-glycopeptides using the synergistic method. Hence the use of combined materials showed an enrichment of about 13 folds for N-linked glycopeptides. These results clearly demonstrate the advantage of the synergistic enrichment method over using one enrichment material alone in detecting glycopeptides, especially in complex biological samples.

## Conclusion

We prepared boronic acid-modified, mesoporous TiO_2_-coated magnetic Fe_3_O_4_ nanoparticles. By combining the B-Fe_3_O_4_@mTiO_2_ with PMMA nanobeads, we have developed a new synergistic method for enriching N-glycopeptides specifically. The coverage of boron moieties onto the Fe_3_O_4_@mTiO_2_ decreased the specific surface area and pore volume greatly, but did not affect the pore size, thereby maintaining the permeability of the enriching material towards glycopeptides. Although the saturation magnetization value of the material was much diminished than that of bare Fe_3_O_4_, the separation of the material by an external magnet could be finished within 30 s, indicating its excellent superparamagnetic property. The results of enriching standard glycopeptides showed that, compared to using B-Fe_3_O_4_@mTiO_2_ alone, the synergistic approach could detect more number of glycopeptides in MS spectra with a cleaner background, and thus the signal-to-noise ratio increased dramatically and the sensitivity improved greatly. The combined materials also outperformed the commercial SiMAG-boronic acid particles in the identification of more glycopeptides from standard samples. The recovery of glycopeptides by the synergistic enrichment method was measured to be 92.1%, which is the highest in the literature. Finally the method was applied to detect N-glycopeptides in amniotic fluid with obtaining the maximum number and ratio. We therefore anticipate a high potential for the use of this method in analyzing glycopeptides in biological samples.

## Electronic supplementary material

Supporting Information
