# Supplementary material for: Boronic Acid-Modified Magnetic Fe3O4@mTiO2 Microspheres for Highly Sensitive and Selective Enrichment of N-Glycopeptides in Amniotic Fluid
Source: Sci Rep. 2017 Jul 4;7:4603. doi: 10.1038/s41598-017-04517-8 (PMC5496847; doi:10.1038/s41598-017-04517-8)
Supplement: Supplementary file 1 — Supporting Information [file 41598_2017_4517_MOESM1_ESM.pdf]

Electronic Supplementary Information for

Boronic acid-modified magnetic Fe_3O_4@mTiO_2 microspheres for highly sensitive and selective enrichment of N-glycopeptides in amniotic fluid

Zhonghua Shi,[1#] Liyong Pu,[2#] Yueshuai Guo,[1#] Ziyi Fu,[1] Wene Zhao,[1] Yunxia Zhu,[1]* Jindao Wu,[2]* Fuqiang Wang[1]*

[1] State Key Laboratory of Reproductive Medicine, Department of Biochemistry and Molecular Biology of Nanjing Medical University, Nanjing Maternity and Child Health Care Hospital Affiliated to Nanjing Medical University, Nanjing, Jiangsu 210029, China

[2] Key Laboratory of Living Donor Liver Transplantation, National Health and Family Planning Commission of the People's Republic of China; Department of Liver Transplantation Center, The First Affiliated Hospital of Nanjing Medical University, Nanjing, China

[#] Zhonghua Shi, Liyong Pu, Yueshuai Guo contributed equally to this work.

* Correspondence to:

Yunxia Zhu: zhuyx@njmu.edu.cn;

Jindao Wu: wujindao@njmu.edu.cn;

Fuqiang Wang: wangfq@njmu.edu.cn

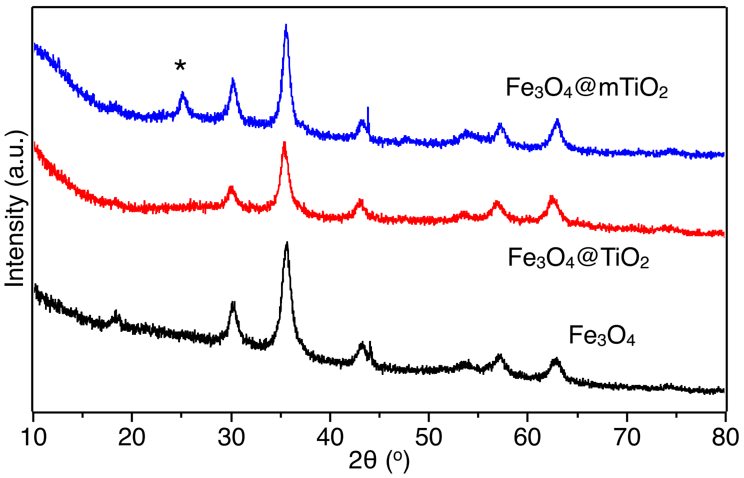

Fig. S1 XRD patterns of Fe_3O_4, Fe_3O_4@TiO_2, and Fe_3O_4@mTiO_2. The asterisk located near 25° indicates the (101) plane of anatase-phase TiO_2.

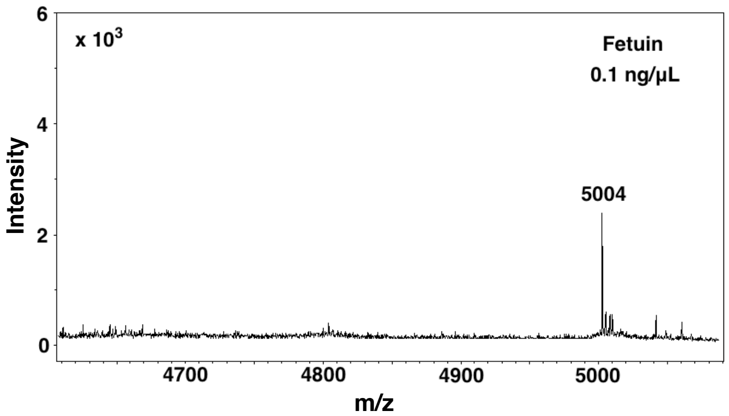

Fig. S2 MALDI-TOF/TOF mass spectra of the tryptic digest of fetuin after enrichment by a synergy of B-Fe_3O_4@mTiO_2 microspheres and PMMA nanobeads.

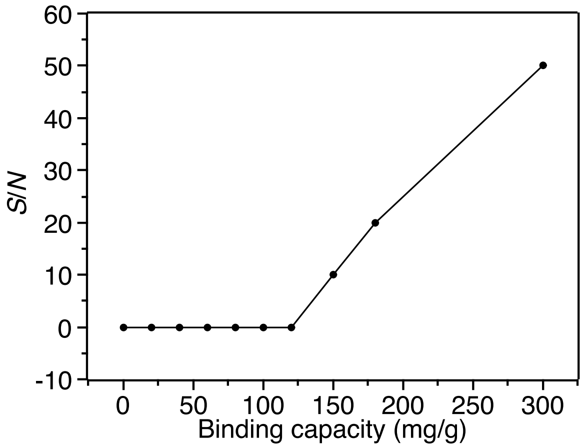

Fig. S3 Binding capacity analysis of the present approach by MALDI-TOF/TOF MS.

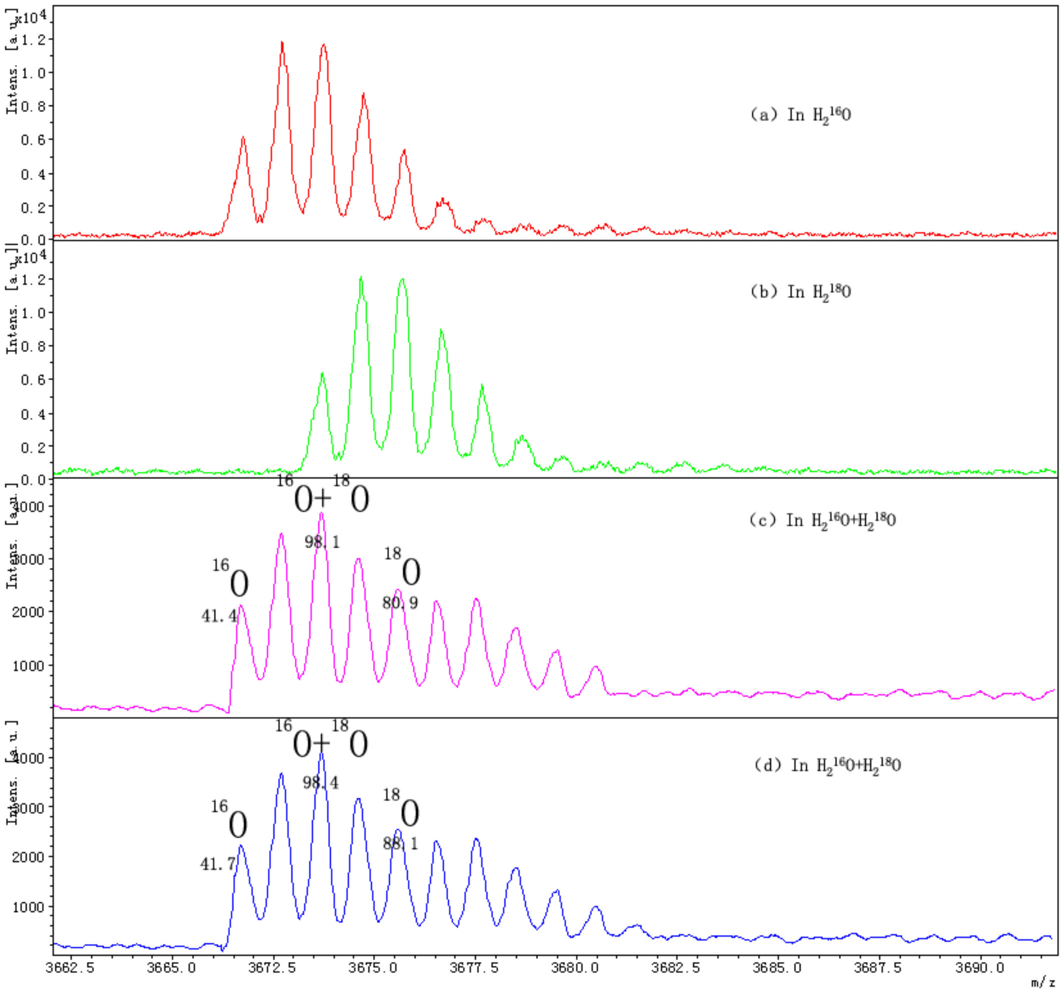

Fig. S4 MALDI-TOF/TOF MS of glycopeptides m/z=3671 GLIQSDQELFSSPN#ATDTIPLVR (a mixture of ^{18}O-labeled enriched and an equal amount of unlabeled unenriched, N# denotes the N-linked glycosylation site). The mass spectra of this glycopeptide (a) labeled by ^{16}O, (b) 100% labeled by ^{18}O, (c) enriched by B-Fe_3O_4@mTiO_2microspheres and (d) enriched by a combination of B-Fe_3O_4@mTiO_2 microspheres and PMMA nanobeads.

Table S1 Detailed information of the observed glycopeptides obtained from tryptic HRP.

Peak number	Observed m/z	Glycan composition	Amino acid sequence
No.1	1895	XylMan3FucGlcNAc2	LHFHDCFVNGCDASILLDN#TTSFR
No.2	2074	XylMan3GlcNAc2	PN#VSNIVRRR
No.3	2445	XylMan3GlcNAc2	PTLN#TTYLQTLR
No.4	2591	XylMan3FucGlcNAc2	PTLN#TTYLQTLR
No.5	2850	FucGlcNAc	GLIQSDQELFSSPN#ATDTIPLVR
No.6	3041	XylMan2GlcNAc2	SFAN#STQTFFNAFVEAMDR
No.7	3353	XylMan3FucGlcNAc2	SFAN#STQTFFNAFVEAMDR
No.8	3525	XylMan3GlcNAc2	GLIQSDQELFSSPN#ATDTIPLVR
No.9	3671	XylMan3FucGlcNAc2	GLIQSDQELFSSPN#ATDTIPLVR
No.10	3895	XylMan3FucGlcNAc2	LHFHDCFVNGCDASILLDN#TTSFR
No.11	4223	XylMan3FucGlcNAc2	QLTPTFYDNSC(AAVESACPR)PN#VSNIVR
No.12	4983	XylMan3FucGlcNAc2<br>XylMan3FucGlcNAc2	LYN#FSNTGLPDPTLN#TTYLQTLR

Table S2 Parameters for the calculation of recovery according to the equation below:

$$\frac{1}{\text{Recovery}} = \text{Ratio}(^{16}O/^{18}O) = \frac{I_0}{I_2 + I_4 - (M_2/M_0)I_2 - [M_2/M_0 + M_4/M_0 - (M_2/M_0)^2]I_0}$$

where M_0, M_2 and M_4 are the theoretical relative intensities of the isotopic envelope of the glycopeptide, which are 3671.7 (51.43%), 3673.7 (100.00%) and 3675.7 (41.09%) respectively, calculated using MS-Isotope (http://prospector.ucsf.edu). I_0, I_2 and I_4 are the actual measured relative intensities of the monoisotope peak for the glycopeptide, the peak with 2 Da increase in mass, and the peak with 4 Da increase in mass, respectively.

		Relative Intensity		
	m/z	Theoretical	B-Fe_3O_4@$mTiO_2$	B-Fe_3O_4@$mTiO_2$ + PMMA
I_0	3671.7		41.4	41.7
I_2	3673.4		98.1	98.4
I_4	3675.7		80.9	88.1
M_0	3671.7	51.43		
M_2	3673.7	100		
M_4	3675.7	41.09		
M_2/M_0		1.944		
M_4/M_0		0.800		
$(M_2/M_0)^2$		3.780		
$M_2/M_0+M_4/M_0-(M_2/M_0)^2$		-1.036		
Ratio($^{16}O/^{18}O$)			1.328	1.086
Recovery(%)			75.3	92.1

Table S3 List of identified glycoproteins from 1 μL of human amniotic fluid by using different enrichment methods after three parallel runs. #N denotes the N-linked glycosylation site. Note: E1 corresponds to using SiMAG-boronic acid particles; E2 using B-Fe_3O_4@mTiO_2 microspheres; and E3 using a combination of B-Fe_3O_4@mTiO_2 and PMMA.

Method	No.	First proteins	Gene name	Description	Modified peptides
E1	1	A0A0G2JRN3	SERPINA1	Alpha-1-antitrypsin	YLG#NATAIFFLPDEGK
E1	2	A0A087WSY5	CPB2	Carboxypeptidase B2	QVHFFV#NASDVDNVK
E1	3	E9PJ21	HYOU1	Hypoxia up-regulated protein 1 (Fragment)	VFGSQ#NLTTVK
E1	4	J3KNI6	ITGB2	Integrin beta (Fragment)	LT#NNSNQFQTEVGK
E1	5	A0A087WXM8	BCAM	Basal cell adhesion molecule	TQ#NFTLLVQGSPELK
E1	6	A9JR48	SALL3	SALL3 protein	SHTGERPFKC#NICGNR
E1	7	A0A087WY93	SERPINA3	Alpha-1-antichymotrypsin (Fragment)	YTG#NASALFILPDQDK
E1	8	H0Y991	COL12A1	Collagen alpha-1(XII) chain (Fragment)	MLEAY#NLTEK
E1	9	A0A087X0S5	COL6A1	Collagen alpha-1(VI) chain	ENYAELLEDAFLK#NVTAQICIDK
E1	10	A0A0A0MT20	EMILIN1	EMILIN-1 (Fragment)	ET#NTTSQMQAALLEK
E1	11	Q30118	HLA-DRA	HLA class II histocompatibility antigen, DR alpha chain	FTPPVV#NVTWLR
E1	12	X5D2U9	HLA-DRB4	HLA class II histocompatibility antigen, DR beta 4 chain	CECHFL#NGTER
E1	13	A0A0G2JNZ5	GBA	Glucosylceramidase	TYTYADTPDDFQLH#NFSLPEEDTK
E1	14	A0A0G2JMB2	IGHA2	Ig alpha-2 chain C region (Fragment)	LAGKPTHV#NVSVVMAEVDGTCY
E1					TPLTA#NITK
E1	15	A0A0G2J	HLA-DRB1	HLA class II	CHFF#NGTER

		NX0		histocompatibility antigen, DRB1-16 beta chain	
E1	16	A0A0J9YYJ1	PON2	Serum paraoxonase/arylesterase 2 (Fragment)	HTNM#NLTQLK
E1	17	A6PWM2	CRELD2	Cysteine-rich with EGF-like domain protein 2 (Fragment)	#NETHSICTACDESCK
E1	18	A8K2M3	WFDC2	WAP four-disulfide core domain 2, isoform CRA_c	TGVCPELQADQ#NCTQECVSDSECADNLK
E1	19	H3BLV0	CD55	Complement decay-accelerating factor (Fragment)	GSQWSDIEEFC#NR
E1	20	B4DNG0	OLFML3	Olfactomedin-like protein 3	IYVLDGTQ#NDTAFVFPR
E1	21	C9JEU5	FGG	Fibrinogen gamma chain	VDKDLQSLEDILHQVE#NK
E1	22	C9JEV0	AZGP1	Zinc-alpha-2-glycoprotein	DIVEYY#NDSNGSHVLQGR
E1					DIVEYYNDS#NGSHVLQGR
E1	23	C9JIZ6	PSAP	Prosaposin	T#NSTFVQALVEHVKEECDR
E1	24	C9JV77	AHSG	Alpha-2-HS-glycoprotein	VCQDCPLLAPL#NDTR
E1	25	C9JYY6	NRCAM	Neuronal cell adhesion molecule	VNVV#NSTLAEVHWDPVPLK
E1	26	Q5JPC9	DKFZp667H216	ABI gene family, member 3 (NESH) binding protein, isoform CRA_d	QVIQ#NVTHK
E1					VHI#NTTSDSILLK
E1	27	D6R9X8	ITGA3	Integrin alpha-3	ELAVPDGYT#NR
E1	28	D6RIS9	SEPP1	Selenoprotein P (Fragment)	CG#NCSLTTLKDEDFCK
E1	29	E5RGB0	CLU	Clusterin (Fragment)	KKEDAL#NETR
E1	30	Q5JWQ4	NRP1	Neuropilin-1	RGPECSQ#NYTTPSGVIK
E1	31	E9PFZ2	CP	Ceruloplasmin	ELHHLQEQ#NVSNAFLDK
E1	32	E9PJ35	SSR2	Translocon-associa	IAPAS#NVSHTVVLRPLK

				ted protein subunit beta	
E1	33	E9PKA3	EFEMP2	EGF-containing fibulin-like extracellular matrix protein 2 (Fragment)	CQCEPGFQLGPN#NR
E1	34	E9PKC6	CD44	CD44 antigen	AF#NSTLPTMAQMEK
E1	35	F5H4C6	GNS	N-acetylglucosamine-6-sulfatase (Fragment)	YPHNHHVV#NNTLEGNCSSK
E1					YPHNHHVVNNTLEG#NCSSK
E1	36	F8WAJ0	DDX31	Probable ATP-dependent RNA helicase DDX31	I#NVSEIK
E1	37	G3V1K2	TMEM5	Transmembrane protein 5	MKFTNILESSFLMN#NKS
E1	38	G3V511	LTBP2	Latent-transforming growth factor beta-binding protein 2	DECWCPA#NSTGK
E1					NVCGGQCCPGWTTA#NSTNHCIKPVCEPPCQNR
E1					NVCGGQCCPGWTTANST#NHCIKPVCEPPCQNR
E1	39	H0Y827	FKBP10	Peptidyl-prolyl cis-trans isomerase FKBP10 (Fragment)	TLSRPSETC#NETTK
E1	40	H0Y950	CD38	ADP-ribosyl cyclase/cyclic ADP-ribose hydrolase 1 (Fragment)	IFDK#NSTFGSVEVHNLQPEK
E1	41	H0YBZ2	CD74	HLA class II histocompatibility antigen gamma chain (Fragment)	YG#NMTEDHVMHLLQNADPLK
E1	42	H3BMA1	MSLN	Mesothelin (Fragment)	KW#NVTSLETLK
E1	43	H3BN02	ITGAX	Integrin alpha-X	YL#NFSESEEK
E1	44	H3BS21	HP	Haptoglobin	NLFL#NHSENATAK

				(Fragment)	
E1					NLFLNHSE#NATAK
E1					VVLHP#NYSQVDIGLIK
E1	45	P02675	FGB	Fibrinogen beta chain	GTAGNALMDGASQLMGE#NR
E1	46	P02787	TF	Serotransferrin	CGLVPVLAENY#NK
E1					QQQHLFGS#NVTDCSGNFCLFR
E1					QQQHLFGSNVTDCSG#NFCLFR
E1	47	P02790	HPX	Hemopexin	ALPQPQ#NVTSLLGCTH
E1	48	P04196	HRG	Histidine-rich glycoprotein	HSHN#NNSSDLHPHK
E1					HSHNN#NSSDLHPHK
E1	49	P06756	ITGAV	Integrin alpha-V	A#NTTQPGIVEGGQVLK
E1	50	P07585	DCN	Decorin	IADT#NITSIPQGLPPSLTELHLDGNK
E1	51	P08603	CFH	Complement factor H	IPCSQPPQIEHGTI#NSSR
E1	52	P11047	LAMC1	Laminin subunit gamma-1	LLN#NLTSIK
E1	53	P30533	LRPAP1	Alpha-2-macroglobulin receptor-associated protein	VIDLWDLAQSA#NLTDKELEAFREELK
E1	54	P35555	FBN1	Fibrillin-1	#NCTDIDECR
E1					AWGTPCEMCPAV#NTSEYK
E1	55	P43652	AFM	Afamin	DIENF#NSTQK
E1	56	P51888	PRELP	Prolargin	I#NGTQICPNDLVAFHDFSSDLENVPHLR
E1	57	P98160	HSPG2	Basement membrane-specific heparan sulfate proteoglycan core protein	SLTQGSLIVGDLAPV#NGTSQGK
E1	58	Q4G0W3	COL14A1	COL14A1 protein	VVDRG#NGSRPSSPEEVK
E1	59	Q07954	LRP1	Prolow-density lipoprotein receptor-related protein 1	L#NGTDPIVAADSK
E1					RGSKD#NATDSVPLR

E1	60	Q08380	LGALS3BP	Galectin-3-binding protein	AAIPSALDT#NSSK
E1					DAGVVCT#NETR
E1	61	Q12797	ASPH	Aspartyl/asparaginyl beta-hydroxylase	LVQLFP#NDTSLK
E1	62	Q86X91	PTK7	Inactive tyrosine-protein kinase 7	DGTPLSDGQS#NHTVSSK
E1	63	Q14956	GPNMB	Transmembrane glycoprotein NMB	NDR#NSSDETFLK
E1	64	Q8IUX7	AEBP1	Adipocyte enhancer-binding protein 1	GVVTDEQGIPIA#NATISVSGINHGVK
E1	65	Q99538	LGMN	Legumain	SHT#NTSHVMQYGNK
E1					SHTNTSHVMQYG#NK
E1	66	Q9UGT4	SUSD2	Sushi domain-containing protein 2	SELV#NETR
E2	1	A0A0G2JRN3	SERPINA1	Alpha-1-antitrypsin	YLG#NATAIFFLPDEGK
E2	2	A0A087WWA5	TNXB	Tenascin-X	RPWG#NLTAELSR
E2	3	E9PJ21	HYOU1	Hypoxia up-regulated protein 1 (Fragment)	E#NGTDTVQEEEESPAEGSK
E2					VFGSQ#NLTTVK
E2	4	A0A087WWL9	HLA-C	HLA class I histocompatibility antigen, Cw-6 alpha chain	GYY#NQSEAGSHTLQR
E2	5	J3KNI6	ITGB2	Integrin beta (Fragment)	LT#NNSNQFQTEVGK
E2	6	A0A087WXM8	BCAM	Basal cell adhesion molecule	TQ#NFTLLVQGSPELK
E2	7	A0A087WY93	SERPINA3	Alpha-1-antichymotrypsin (Fragment)	YTG#NASALFILPDQDK
E2	8	A0A087X0S5	COL6A1	Collagen alpha-1(VI) chain	ENYAELLEDAFLK#NVTAQICIDK
E2					R#NFTAADWGQSR
E2	9	A0A0A0MT20	EMILIN1	EMILIN-1 (Fragment)	ET#NTTSQMQAALLEK
E2					LGAL#NSSLQLLEDR
E2	10	Q30118	HLA-DRA	HLA class II	FTPPVV#NVTWLR

				histocompatibility antigen, DR alpha chain	
E2	11	A0A0G2JJV3	HLA-DPB1	HLA class II histocompatibility antigen, DP beta 1 chain	QECYAF#NGTQR
E2	12	X5D2U9	HLA-DRB4	HLA class II histocompatibility antigen, DR beta 4 chain	CECHFL#NGTER
E2	13	A0A0G2JMB2	IGHA2	Ig alpha-2 chain C region (Fragment)	TPLTA#NITK
E2	14	O19617	HLA-C	HLA class I antigen	GYY#NQSEDGSHTLQR
E2	15	A8K2M3	WFDC2	WAP four-disulfide core domain 2, isoform CRA_c	TGVCPELQADQ#NCTQECVSDSECADNLK
E2	16	V9GYE7	CFHR2	Complement factor H-related protein 2	LQNNEN#NISCVER
E2	17	B1ALD9	POSTN	Periostin	EV#NDTLLVNELK
E2	18	B4DNG0	OLFML3	Olfactomedin-like protein 3	IYVLDGTQ#NDTAFVFPR
E2	19	C9JEU5	FGG	Fibrinogen gamma chain	VDKDLQSLEDILHQVE#NK
E2	20	C9JEV0	AZGP1	Zinc-alpha-2-glycoprotein	DIVEYY#NDSNGSHVLQGR
E2	21	C9JV77	AHSG	Alpha-2-HS-glycoprotein	VCQDCPLLAPL#NDTR
E2	22	Q5JPC9	DKFZp667H216	ABI gene family, member 3 (NESH) binding protein, isoform CRA_d	QVIQ#NVTHK
E2	23	D6R9X8	ITGA3	Integrin alpha-3	ELAVPDGYT#NR
E2	24	E5RGB0	CLU	Clusterin (Fragment)	KKEDAL#NETR
E2	25	E7ENL6	COL6A3	Collagen alpha-3(VI) chain	Q#NLTVTDR
E2	26	E9PFZ2	CP	Ceruloplasmin	EHEGAIYPD#NTTDFQR
E2					ELHHLQEQ#NVSNAFLDK
E2	27	E9PJ35	SSR2	Translocon-associated protein subunit beta	IAPAS#NVSHTVVLRPLK
E2	28	E9PKC6	CD44	CD44 antigen	AF#NSTLPTMAQMEK

E2	29	F5GZN3	MFGE8	Lactadherin	VAYS#NDSANWTEYQDPR
E2	30	H0Y950	CD38	ADP-ribosyl cyclase/cyclic ADP-ribose hydrolase 1 (Fragment)	IFDK#NSTFGSVEVHNLQPEK
E2	31	H0YBZ2	CD74	HLA class II histocompatibility antigen gamma chain (Fragment)	YG#NMTEDHVMHLLQNADPLK
E2	32	H3BMA1	MSLN	Mesothelin (Fragment)	KW#NVTSLETLK
E2	33	H3BS21	HP	Haptoglobin (Fragment)	NLFL#NHSENATAK
E2					NLFLNHSE#NATAK
E2					VVLHP#NYSQVDIGLIK
E2	34	O15031	PLXNB2	Plexin-B2	SCVAVTSAQPQ#NMSR
E2	35	O43157	PLXNB1	Plexin-B1	YTLDP#NITSAGPTK
E2	36	O60487	MPZL2	Myelin protein zero-like protein 2	VLEAV#NGTDAR
E2	37	Q5H9B4	TIMP1	Metalloproteinase inhibitor 1 (Fragment)	FVGTPEV#NQTTLYQR
E2	38	P02675	FGB	Fibrinogen beta chain	GTAGNALMDGASQLMGE#NR
E2	39	P02763	ORM1	Alpha-1-acid glycoprotein 1	QDQCIY#NTTYLNVQR
E2	40	P02787	TF	Serotransferrin	CGLVPVLAENY#NK
E2					QQQHLFGS#NVTDCSGNFCLFR
E2					QQQHLFGSNVTDCSG#NFCLFR
E2	41	P02790	HPX	Hemopexin	ALPQPQ#NVTSLLGCTH
E2	42	P04196	HRG	Histidine-rich glycoprotein	VIDF#NCTTSSVSSALANTK
E2	43	P06756	ITGAV	Integrin alpha-V	A#NTTQPGIVEGGQVLK
E2	44	P07585	DCN	Decorin	IADT#NITSIPQGLPPSLTELHLDGNK
E2	45	P08603	CFH	Complement factor H	MDGAS#NVTCINSR
E2	46	P14625	HSP90B1	Endoplasmin	HN#NDTQHIWESDSNEFSVIADPR

E2	47	P19652	ORM2	Alpha-1-acid glycoprotein 2	QNQCFY#NSSYLNVQR
E2	48	P30533	LRPAP1	Alpha-2-macroglobulin receptor-associated protein	VIDLWDLAQSA#NLTDKELEAFREELK
E2	49	P32004	L1CAM	Neural cell adhesion molecule L1	GY#NVTYWR
E2	50	P35555	FBN1	Fibrillin-1	#NCTDIDECR
E2					AWGTPCEMCPAV#NTSEYK
E2	51	P43652	AFM	Afamin	DIENF#NSTQK
E2	52	Q4G0W3	COL14A1	COL14A1 protein	VVDRG#NGSRPSSPEEVK
E2	53	Q06828	FMOD	Fibromodulin	LYLDHN#NLTR
E2	54	Q07954	LRP1	Prolow-density lipoprotein receptor-related protein 1	GVTHL#NISGLK
E2					LTSCAT#NASICGDEAR
E2	55	Q08380	LGALS3BP	Galectin-3-binding protein	AAIPSALDT#NSSK
E2					DAGVVCT#NETR
E2	56	Q14956	GPNMB	Transmembrane glycoprotein NMB	NDR#NSSDETFLK
E2	57	Q9P2B2	PTGFRN	Prostaglandin F2 receptor negative regulator	ELDLTC#NITTDR
E2	58	Q9TQE0	HLA-DRB1	HLA class II histocompatibility antigen, DRB1-9 beta chain	QDKFECHFF#NGTER
E2	59	Q9UBG0	MRC2	C-type mannose receptor 2	TS#NISKPGTLER
E2	60	Q9Y5Y6	ST14	Suppressor of tumorigenicity 14 protein	VI#NQTTCENLLPQQITPR
E3	1	A0A0G2JRN3	SERPINA1	Alpha-1-antitrypsin	YLG#NATAIFFLPDEGK
E3	2	A0A087WWA5	TNXB	Tenascin-X	A#NQTYTSVAR
E3					RPWG#NLTAELSR
E3	3	E9PJ21	HYOU1	Hypoxia	E#NGTDTVQEEEESPAE

				up-regulated protein 1 (Fragment)	GSK
E3					VFGSQ#NLTTVK
E3	4	A0A087WWL9	HLA-C	HLA class I histocompatibility antigen, Cw-6 alpha chain	GYY#NQSEAGSHTLQR
E3	5	J3KNI6	ITGB2	Integrin beta (Fragment)	LT#NNSNQFQTEVGK
E3	6	A0A087WXM8	BCAM	Basal cell adhesion molecule	TQ#NFTLLVQGSPELK
E3	7	H0Y991	COL12A1	Collagen alpha-1(XII) chain (Fragment)	MLEAY#NLTEK
E3	8	A0A087X0S5	COL6A1	Collagen alpha-1(VI) chain	R#NFTAADWGQSR
E3	9	A0A0A0MT20	EMILIN1	EMILIN-1 (Fragment)	ET#NTTSQMQAALLEK
E3					LGAL#NSSLQLLEDR
E3	10	A0A0C4DGZ9	TPP1	Tripeptidyl-peptidase 1	Y#NLTSQDVGSGTSNNSQACAQFLEQYFHDSDLAQFMR
E3					YNLTSQDVGSGTS#NNSQACAQFLEQYFHDSDLAQFMR
E3	11	A0A0G2JJV3	HLA-DPB1	HLA class II histocompatibility antigen, DP beta 1 chain	QECYAF#NGTQR
E3	12	X5D2U9	HLA-DRB4	HLA class II histocompatibility antigen, DR beta 4 chain	CECHFL#NGTER
E3	13	A0A0G2JMB2	IGHA2	Ig alpha-2 chain C region (Fragment)	TPLTA#NITK
E3	14	A0A0G2JNY4	HLA-A	HLA class I histocompatibility antigen, A-3 alpha chain	GYY#NQSEAGSHTVQR
E3	15	O19617	HLA-C	HLA class I antigen	GYY#NQSEDGSHTLQR
E3	16	V9GYE7	CFHR2	Complement factor H-related protein 2	LQNNEN#NISCVER
E3	17	B1ALD9	POSTN	Periostin	EV#NDTLLVNELK

E3	18	B4DNG0	OLFML3	Olfactomedin-like protein 3	IYVLDGTQ#NDTAFVFPR
E3	19	C9JA36	ATP1B3	Sodium/potassium-transporting ATPase subunit beta-3 (Fragment)	#NLTVCPDGALFEQK
E3	20	C9JEU5	FGG	Fibrinogen gamma chain	VDKDLQSLEDILHQVE#NK
E3	21	C9JEV0	AZGP1	Zinc-alpha-2-glycoprotein	DIVEYY#NDSNGSHVLQGR
E3	22	C9JIZ6	PSAP	Prosaposin	#NSTKQEILAALEK
E3					DVVTAAGDMLKD#NATEEEILVYLEK
E3					T#NSTFVQALVEHVKEECDR
E3					TCDWLPKP#NMSASCK
E3	23	C9JV77	AHSG	Alpha-2-HS-glycoprotein	VCQDCPLLAPL#NDTR
E3	24	Q5JPC9	DKFZp667H216	ABI gene family, member 3 (NESH) binding protein, isoform CRA_d	QVIQ#NVTHK
E3	25	D6R9X8	ITGA3	Integrin alpha-3	ELAVPDGYT#NR
E3	26	D6RHJ6	JCHAIN	Immunoglobulin J chain (Fragment)	E#NISDPTSPLR
E3	27	E5RGB0	CLU	Clusterin (Fragment)	KKEDAL#NETR
E3	28	E7ENL6	COL6A3	Collagen alpha-3(VI) chain	Q#NLTVTDR
E3	29	E9PFZ2	CP	Ceruloplasmin	EHEGAIYPD#NTTDFQR
E3					ELHHLQEQ#NVSNAFLDK
E3	30	E9PJ35	SSR2	Translocon-associated protein subunit beta	IAPAS#NVSHTVVLRPLK
E3	31	E9PKC6	CD44	CD44 antigen	AF#NSTLPTMAQMEK
E3	32	G3XAK1	MST1	Hepatocyte growth factor-like protein	GTA#NTTTAGVPCQR
E3	33	H0Y950	CD38	ADP-ribosyl cyclase/cyclic ADP-ribose hydrolase 1 (Fragment)	IFDK#NSTFGSVEVHNLQPEK
E3	34	H0YBZ2	CD74	HLA class II	YG#NMTEDHVMHLLQNA

				histocompatibility antigen gamma chain (Fragment)	DPLK
E3	35	H0YCY8	CTSC	Dipeptidyl peptidase 1 (Fragment)	VTTYC#NETMTGWVHDVLGR
E3	36	H3BMA1	MSLN	Mesothelin (Fragment)	KW#NVTSLETLK
E3	37	H3BN02	ITGAX	Integrin alpha-X	YL#NFSESEEK
E3	38	H3BS21	HP	Haptoglobin (Fragment)	NLFL#NHSENATAK
E3					NLFLNHSE#NATAK
E3					VVLHP#NYSQVDIGLIK
E3	39	J3QRN2	APOH	Beta-2-glycoprotein 1 (Fragment)	VYKPSAG#NNSLYR
E3	40	O60449	LY75	Lymphocyte antigen 75	QTLQ#NASETVK
E3	41	O60487	MPZL2	Myelin protein zero-like protein 2	VLEAV#NGTDAR
E3	42	Q5H9B4	TIMP1	Metalloproteinase inhibitor 1 (Fragment)	FVGTPEV#NQTTLYQR
E3	43	P02675	FGB	Fibrinogen beta chain	GTAGNALMDGASQLMGE#NR
E3	44	P02763	ORM1	Alpha-1-acid glycoprotein 1	QDQCIY#NTTYLNVQR
E3	45	P02787	TF	Serotransferrin	CGLVPVLAENY#NK
E3					QQQHLFGS#NVTDCSGNFCLFR
E3	46	P02790	HPX	Hemopexin	ALPQPQ#NVTSLLGCTH
E3	47	P04196	HRG	Histidine-rich glycoprotein	VIDF#NCTTSSVSSALANTK
E3	48	P05543	SERPINA7	Thyroxine-binding globulin	VTACHSSQP#NATLYK
E3	49	P06756	ITGAV	Integrin alpha-V	A#NTTQPGIVEGGQVLK
E3	50	P07585	DCN	Decorin	IADT#NITSIPQGLPPSLTELHLDGNK
E3					LGLSFNSISAVD#NGSLANTPHLR
E3	51	P08603	CFH	Complement factor H	MDGAS#NVTCINSR
E3	52	P14625	HSP90B1	Endoplasmin	HN#NDTQHIWESDSNEFSVIADPR

E3	53	P19652	ORM2	Alpha-1-acid glycoprotein 2	QNQCFY#NSSYLNVQR
E3	54	P35555	FBN1	Fibrillin-1	#NCTDIDECR
E3					AWGTPCEMCPAV#NTSEYK
E3	55	P43652	AFM	Afamin	DIENF#NSTQK
E3	56	Q4G0W3	COL14A1	COL14A1 protein	VVDRG#NGSRPSSPEEVK
E3	57	Q06828	FMOD	Fibromodulin	LYLDHN#NLTR
E3	58	Q07954	LRP1	Prolow-density lipoprotein receptor-related protein 1	GVTHL#NISGLK
E3					LTSCAT#NASICGDEAR
E3	59	Q08380	LGALS3BP	Galectin-3-binding protein	AAIPSALDT#NSSK
E3					DAGVVCT#NETR
E3	60	Q86X91	PTK7	Inactive tyrosine-protein kinase 7	DGTPLSDGQS#NHTVSSK
E3	61	Q92673	SORL1	Sortilin-related receptor	LTIV#NSSVLDRPR
E3	62	Q9TQE0	HLA-DRB1	HLA class II histocompatibility antigen, DRB1-9 beta chain	QDKFECHFF#NGTER
E3	63	Q9UBG0	MRC2	C-type mannose receptor 2	TS#NISKPGTLER
E3	64	Q9Y5Y6	ST14	Suppressor of tumorigenicity 14 protein	VI#NQTTCENLLPQQITPR

Table S4 The number of detected peptides and ratio of glycopeptides in 1 μL of human amniotic fluid by different enrichment methods.

Enriching material	Peptides	Glycopeptides	Ratio	Proteins	Glycoproteins	Ratio
**SiMAG-boronic acid**	254	78	31%	189	66	35%
**B-Fe_3O_4@$mTiO_2$**	185	74	40%	143	60	42%
**B-Fe_3O_4@$mTiO_2$ + PMMA**	185	83	45%	137	64	47%
**Control**	92	3	3.3%	82	3	3.7%
